# Does dual-plating offer superior stability in complex distal femoral fracture management?

**DOI:** 10.1186/s13018-026-06876-3

**Published:** 2026-05-06

**Authors:** Ahmed Naser Elbarbary, Ismail Tawfeek Badr, Emad Badawy, Rasha Yousry Kamel Saleh

**Affiliations:** Department of Orthopedic Surgery, Faculty of Medicine, Menoufia University, Shebin El-Kom, Menoufia, 32511 Egypt

**Keywords:** Distal femoral fractures, dual plating fixation, Neer scoring system, Range of motion, Nonunion

## Abstract

**Background:**

Distal femoral fractures (DFF) present significant challenges in orthopedic surgery because of their complex biomechanical environment and limited bone stock for fixation. Dual plating (DP), involving the application of both lateral and medial plates, has emerged as a promising alternative to enhance construct stability. We hypothesize that distal femoral DP improves union rates and functional outcomes in patients with complex or non-united DFFs.

**Methods:**

This prospective observational study was conducted on 30 cases with DFF who had either complex primary fixation or developed nonunion. Patients were monitored and followed up at regular intervals postoperatively (2 weeks, 6 weeks, 3 months, 6 months, and 12 months). Functional recovery was assessed using the Neer scoring system.

**Results:**

The range of motion (ROM) ranged from 70 to 100° with a mean ± SD of 90.17 ± 10.3°. The majority of patients (66.67%) achieved an excellent outcome, and 20% had a good result. Only a small proportion had fair (6.67%) or poor (6.67%) outcomes. There were no cases of malunion or nonunion. However, delayed union and infection were each reported in 3 patients (10%). Patients with excellent outcomes were significantly younger (mean age 24.9 years) and had a lower BMI (24.31 kg/m²) compared to those with poorer outcomes, who were older (≥ 55 years) and had higher BMIs (≥ 28.7 kg/m²).

**Conclusions:**

Distal femur dual plating is a viable salvage option for complex primary fixation of complex distal femur fractures. Good to excellent outcomes can be achieved with a low complication rate especially in patients with normal BMI and younger than 55 years of age.

## Introduction

Distal femoral fractures (DFF), particularly those complicated by nonunion or failed primary fixation, present significant challenges in orthopedic surgery due to their complex biomechanical environment and limited bone stock for fixation. These fractures often involve comminuted metaphyseal and intra-articular components, making stable fixation difficult and increasing the risk of complications such as varus collapse and implant failure [1–4].

**Fig. 1:**
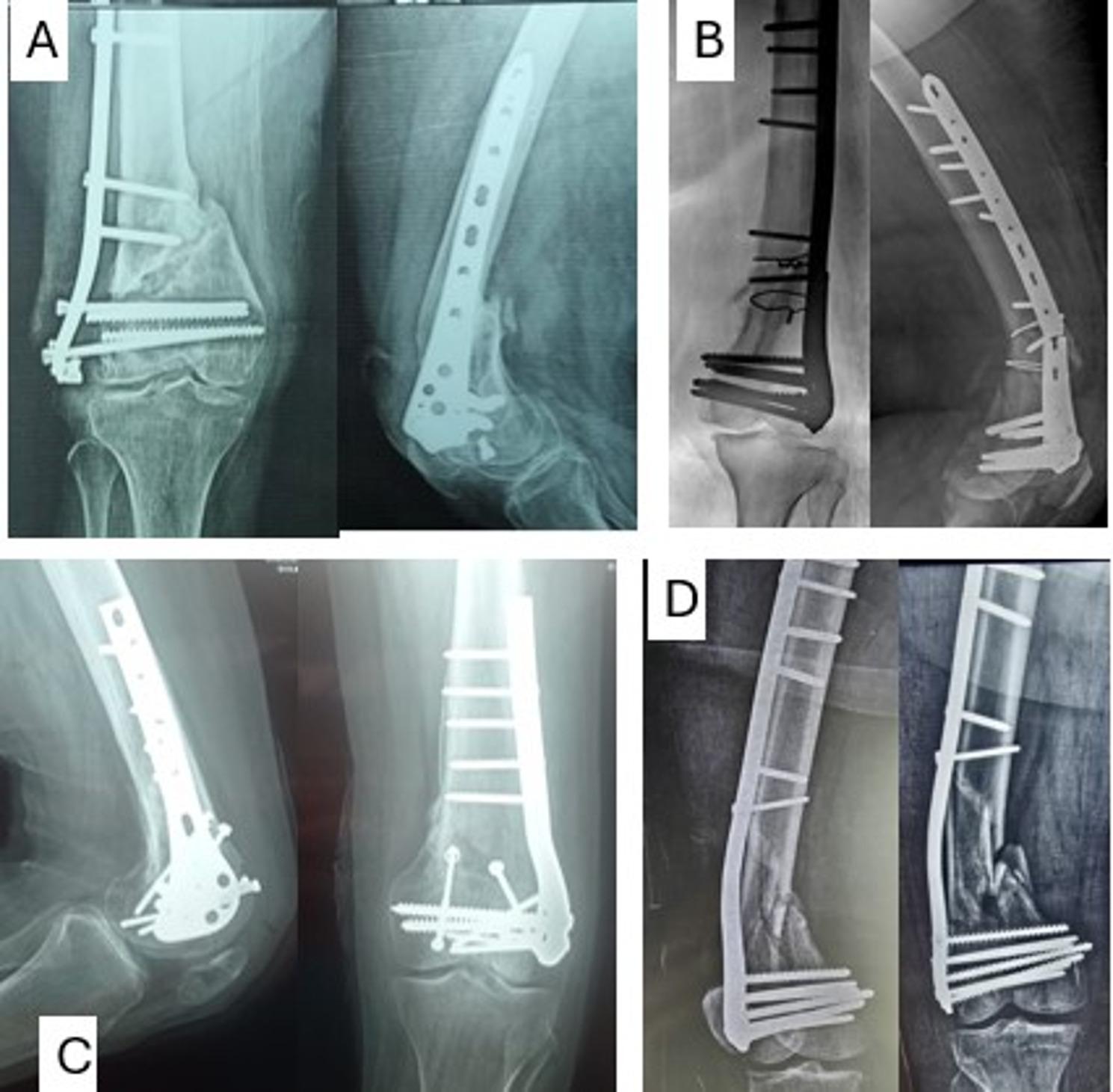
Examples of failure modes or complications encountered with a single lateral column plate construct: **A**; metal failure, fracture nonunion, and screw breakage. **B**: broken plate and nonunion of the fracture. **C**. Healed fracture with varus position. **D**; plastic deformation with a bent plate and nonunion of the fracture

Traditional fixation methods, including lateral locking plates and retrograde intramedullary nailing, have shown limitations in managing these complex fractures, especially in osteoporotic bone or cases with significant comminution. These techniques may not provide sufficient stability, leading to higher rates of varus collapse, malunion, nonunion, and mechanical failure [3, 5–7]. (Fig. 1)

**Fig. 4:**
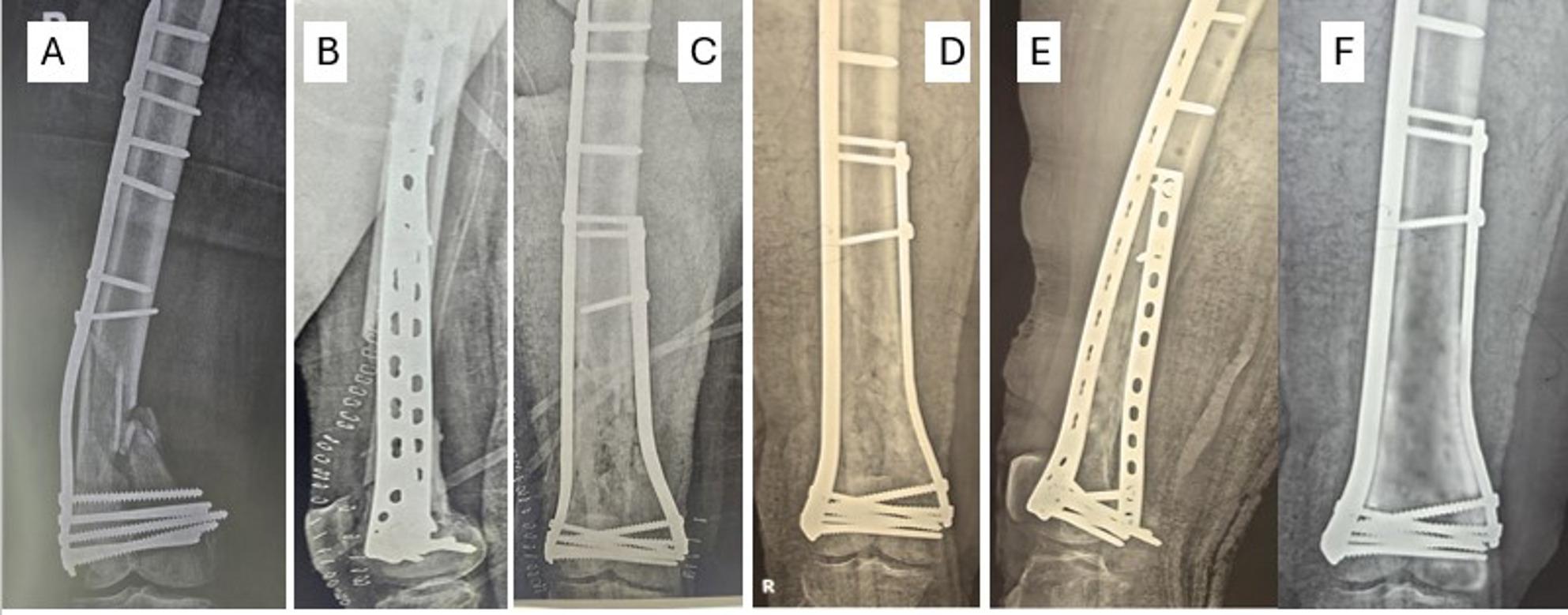
After the patient (Fig. 3) sustained a bent plate **A**, revision was done by dual plating (locked DFP and contoured narrow DCP), early postoperative radiographs **B**, **C**, follow-up at 3 months **D**, **E**, and 16-month follow-up with good consolidation of the fracture **F**

Dual plating (DP), involving the application of both lateral and medial plates, has emerged as a promising alternative to enhance construct stability [3, 4, 8]. Biomechanical investigations have demonstrated that DP offers elevated axial and rotational stiffness, reducing the likelihood of implant failure and promoting fracture healing [2, 9, 10]. DP constructs are mechanically stronger than other constructs commonly used in the treatment of distal femur fractures. Orthogonal and adjacent dual plating are appropriate operative techniques for femoral shaft fracture fixation. Locking plates allow higher construct rigidity and stability compared to conventional dynamic compression plates [9]. This can be done in a minimally invasive fashion, as the medial distal femoral anatomy allows for safe application of DP constructs, giving a solution for patients with distal femur fractures with increased risk for instability, varus collapse, or nonunion [10]. Clinically, DP has been associated with higher rates of union and improved functional results in complex DFF cases [3, 4, 11]. The torsional stiffness of a dual plate construct was 2.6 times that of the single lateral plating and 5.4% higher than that of the intramedullary nail [12].

Despite these advantages, DP is associated with prolonged time in operation and increased blood loss intraoperatively compared to single plating techniques. However, the potential benefits in terms of enhanced stability and reduced nonunion rates may outweigh these drawbacks, particularly in challenging cases [1, 3, 6, 11].

Currently, there is a wider indication for the use of dual plating to achieve a more stable construct, and surgeons are becoming more familiar with different techniques for application. This study aims to evaluate the results of distal femoral DP fixation in primary complex or non-united DFF and assessing the factors that led to satisfactory results versus poor results and complications and comparing the results to those in the literature .

## Patients and methods

This prospective observational study was performed on 30 patients with DFF who were admitted to the orthopaedic department and who had either complex primary fixation or developed nonunion during the period from January 2022 to December 2024 and underwent revision surgery using dual plating fixation of the distal femur fracture, with a minimum one-year follow-up from the dual plating surgery.

Informed and written consent was taken from all patients who were included in the study. The investigation was conducted after approval by the Ethical Committee of Menoufia University Hospitals.

Patients older than 18 years old with radiologically confirmed non-union or complex primary fixation (broken implants and/or screws, loss of alignment or reduction, screw migration) of DFF who were fit for surgery and received a final DP construct were included in the study. Closed fractures of the distal femur or Gustilo-Anderson (type 1, 2) open fractures at the time of the primary injury were included. Open fractures with a significant degree of metaphyseal comminution and displacement, a lengthy interval between the initial fracture and the transition from the temporary external fixation to the definitive fixation, or those that were primarily operated on in a different center are considered complex.

While patients with pathological fractures, open fractures (Gustilo-Anderson type 3) with extensive soft tissue loss, patients unfit for surgery because of systemic disease, pre-existing vascular or neurological injuries, or fractures with active infection were excluded from the study. Patients who had revision surgery with single lateral plating were not included in the study.

## Preoperative assessment

The clinical evaluation of each patient was comprehensive, including a physical examination and history-taking. The baseline data recorded included age, sex, body mass index (BMI), any comorbidities, and the side of the injury.

Radiographs (anteroposterior and lateral views) of the femur were taken to confirm the diagnosis and assess fracture characteristics.

**Fig. 5:**
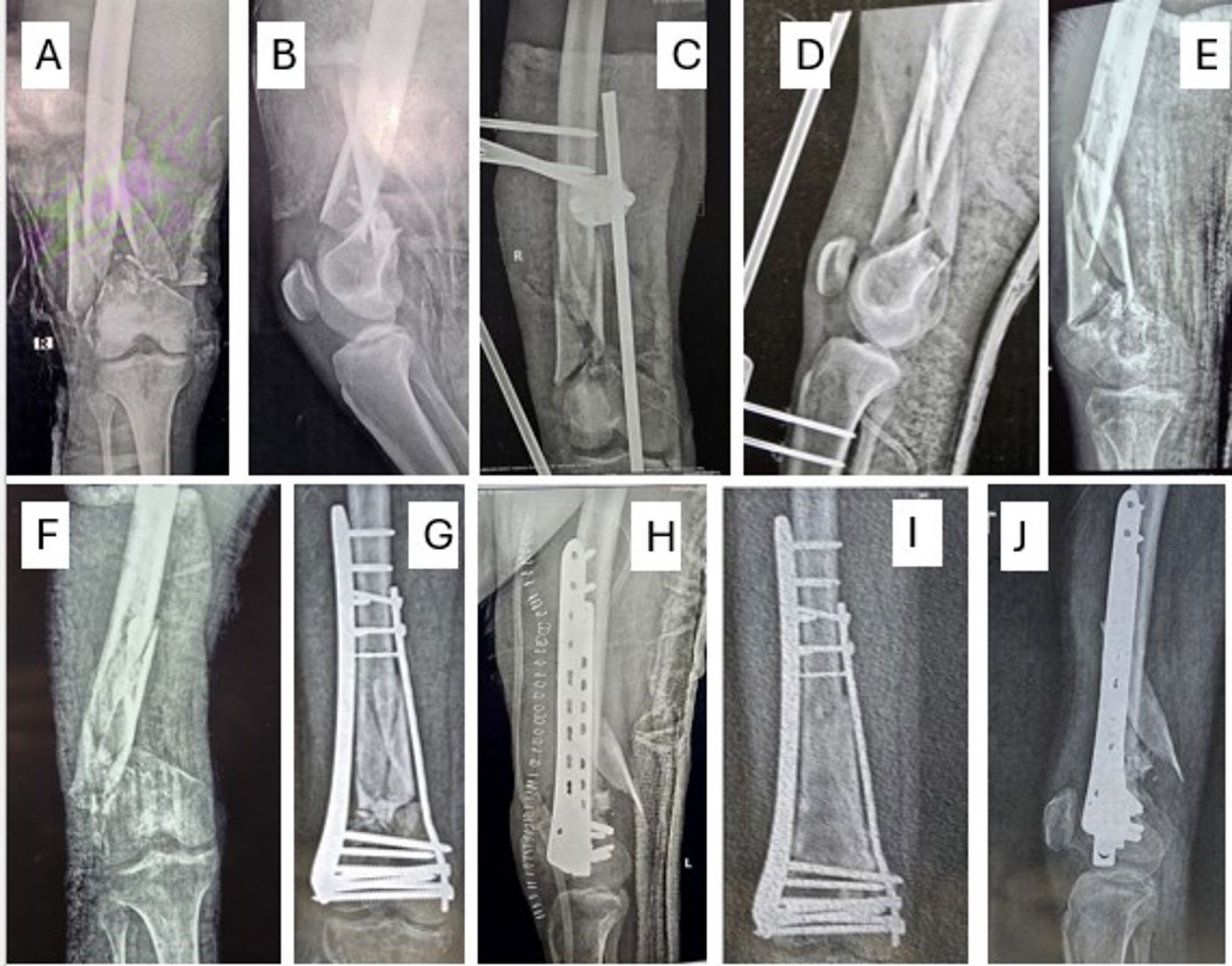
A male patient, 45 years old, presented with an open 1st-degree fracture of the distal femur **A**, **B**. Application of a spanning external fixator was done **C**, **D**. 2 weeks later, the fixator was removed, and the patient was splinted for another 10 days **E**, **F** before the definitive surgery with DFP and contoured medial narrow DCP **G**, **H**. Follow-up at 18 months **I**, **J**

Patients with open fractures were primarily treated for the open wound and a knee-spanning external fixator, and definitive fixation was planned in a staged manner when the wound condition improved. (Fig. 5)

Primary x-ray evaluation showed the use of an above-knee slab was applied to immobilize the affected limb, and it continued in the early postoperative period after the primary intervention. Computerized tomography was also done in order to identify the fracture and comminution and to help plan the surgical approach and intervention. Infection was excluded before the surgery through laboratory investigations and lack of clinical signs of infection.

### Surgical technique

After optimization of the patients and ensuring fitness for anesthesia, all surgical procedures were performed under either general or regional anesthesia, with the patient positioned supine on a radiolucent operating table with no tourniquet used in all patients. An image intensifier was positioned on the opposite side of the injured limb.

To provide excellent access to the femoral condyles and distal femoral metaphysis, a standard lateral approach to the distal femur was utilized initially. An additional medial parapatellar or subvastus approach was made for placement of the medial plate when the medial support was compromised or in the case of severe comminution or loss of bone.

After exposure, all fibrous tissue and necrotic bone were debrided at the non-union or failed fixation site. Care was taken to preserve the periosteum where possible to support revascularization. The medullary canal was gently reopened with a hand reamer to encourage bone healing.

Using fluoroscopic guidance, fracture fragments were reduced and aligned. Reduction of the articular surface and distal femur to the metaphysis was, in the meantime, maintained with K-wires and reduction clamps. Definitive fixation was performed using a pre-contoured lateral distal femoral locking compression plate (LCP), bridging the fracture, and was secured with at least three to five bicortical screws near and far from the fracture site, and the gap is filled with autologous bone graft. Once there is any defect after fracture refreshment and reduction ending by fixation, the bone graft is considered, and for long-standing cases with lost fracture viability, bone graft was also considered.

Subsequently, a medial buttress plate (T buttress plate or compression type) was applied to augment stability, particularly in cases with gross metaphyseal comminution, medial cortical defects, bone loss, osteoporotic fractures, or previous failed fixation. The medial plate acted as an anti-glide or anti-collapse construct, enhancing resistance to varus collapse and also decreasing displacement, bending moment, and strain at the fracture site during axial loading [11, 13, 14]. Only four patients received the second plate through the extensile single lateral approach applied at the anteromedial aspect.

After achieving stable fixation and thorough saline irrigation, hemostasis was ensured. Layers were closed in standard fashion, including the vastus lateralis, fascia, subcutaneous tissue, and skin, using absorbable sutures. A drain was inserted and typically removed after 24–48 h.

An above-knee slab was applied to immobilize the affected limb in the early postoperative period with adequate elevation and cold compression.

## Postoperative care and follow-up

All cases received postoperative intravenous antibiotics and thromboprophylaxis as per institutional protocol.

Rehabilitation was individualized as tolerated and depended primarily on fixation stability. Starting with isometric exercises (static quadriceps and hamstring exercises), followed by passive knee range of motion (ROM), then active-assisted knee ROM.

Patients were monitored and followed up at regular intervals postoperatively (2 weeks, 6 weeks, 3 months, 6 months, and 12 months). The following parameters were evaluated: length of hospital stay, clinical time for union (defined by the lack of pain and tenderness at the fracture site with full weight-bearing capability), radiological time for union (confirmed by bridging callus in at least three cortices on orthogonal X-rays), weight-bearing initiation time (based on radiographic healing and surgeon’s assessment), ROM, and any complications, including malunion, delayed union, nonunion, and infection.

Malunion was defined as the healing of a femoral fracture in an anatomically incorrect position. When the thresholds of > 5–10° angulation, > 10° rotation, or > 1–2 cm shortening are exceeded, patients may experience pain, limp, and asymmetry in their gait. The measurement was performed on plain radiographs.

## Functional outcome assessment

Functional recovery was evaluated utilizing the Neer scoring system [15], which includes anatomical alignment (10 points), pain (35 points), function and activities of daily living (30 points), and ROM (25 points). Scores were classified as < 55 as poor, 55–69 as fair, 70–84 as good, and 85–100 as excellent.

### Statistical analysis

SPSS v26 (IBM Inc., Armonk, NY, USA) was employed to conduct the statistical analysis. The normality of the data distribution was assessed using the Shapiro-Wilks test and histograms. The one-way ANOVA test was used to analyze quantitative data, which were presented as mean and standard deviation (SD). The chi-square test was employed to analyze qualitative data, which were presented as frequency and percentages. A P value of less than 0.05 was regarded as statistically significant.

## Results

**Fig. 2:**
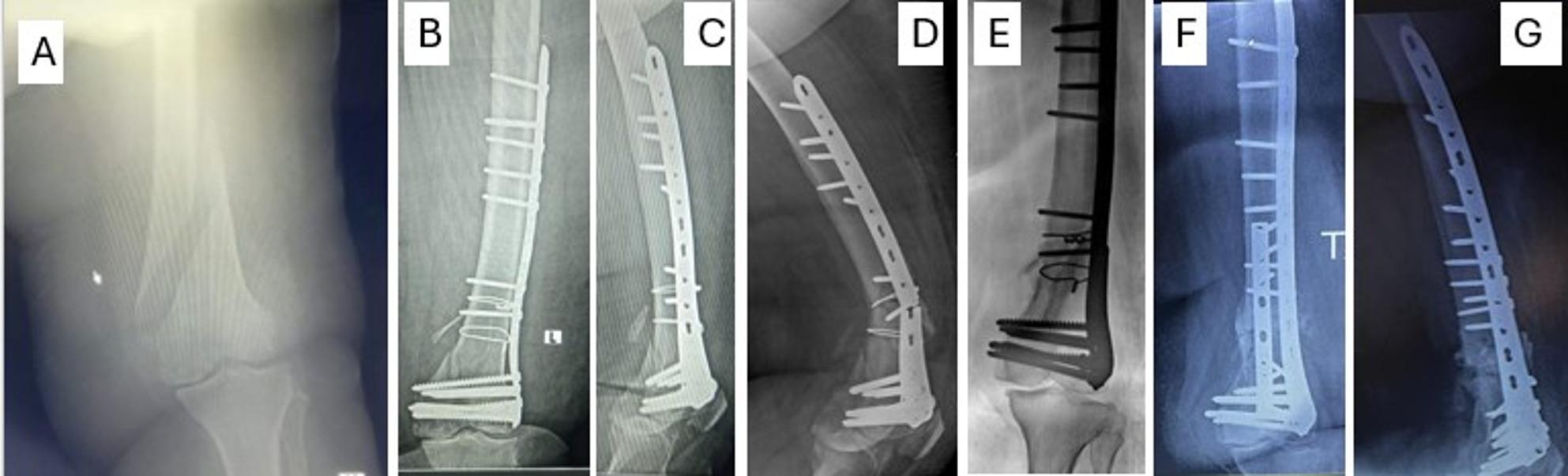
A 72-year-old morbidly obese female patient sustained a left distal femoral fracture **A**; fixation was done with a lateral column locked DFP and cerclage wires **B**, **C**. 2.5 months later, the patient sustained a fall that resulted in a broken plate before full consolidation of the fracture **D**, **E**, which was fixed again with DFP and another additional plate (narrow DCP) to augment the fracture and neutralize forces around fractures. At a follow-up at 1.5 years, the patient was walking independently **F**, **G**

The patients’ ages ranged from 18 to 60 years with a mean ± SD of 32.87 ± 15.01 years. Males were 20 (66.67%), and females were 10 (33.33%). All the fractures included have extensive metaphysical comminution, either AO/OTA 33 type A or C, with high rates of varus collapse with medial comminution. 16 patients have associated comorbidities (7 have hypertension, 5 have DM, 3 have combined hypertension and DM, and one patient had bronchial asthma). The BMI ranged from 20.9 to 29.8 kg/m² with a mean ± SD of 25.65 ± 2.74 kg/m²; 11 patients (36.66%) had primarily open fractures and were treated with spanning external fixators, 6 patients (20%) with bent plates, 7 patients (23.33%) with broken plates, and 6 patients (20%) had loosening at the plate-screw and screw-bone interfaces and established nonunion around the construct. Regarding the injury side, the right side (19 patients) is more commonly affected (63.33%) than the left (36.66%). (Fig. 2)

In terms of the intraoperative data, the total operation time was between 120 and 210 min, with a mean ± SD of 161.17 ± 29.23 min. The blood loss was between 280 and 780 ml, with a mean ± SD of 526.67 ± 164.05 ml. The average hospitalization length was 2.83 ± 0.91 days, with a range of 2 to 4 days. Autogenous iliac crest bone graft was used in 16 patients (at the dual plating surgery) and in another 3 patients with delayed union (as a second-stage surgery). A T buttress plate was used in 17 patients, a contoured narrow DCP was used in 12 patients, and another distal femoral plate was used in one patient.

**Fig. 3:**
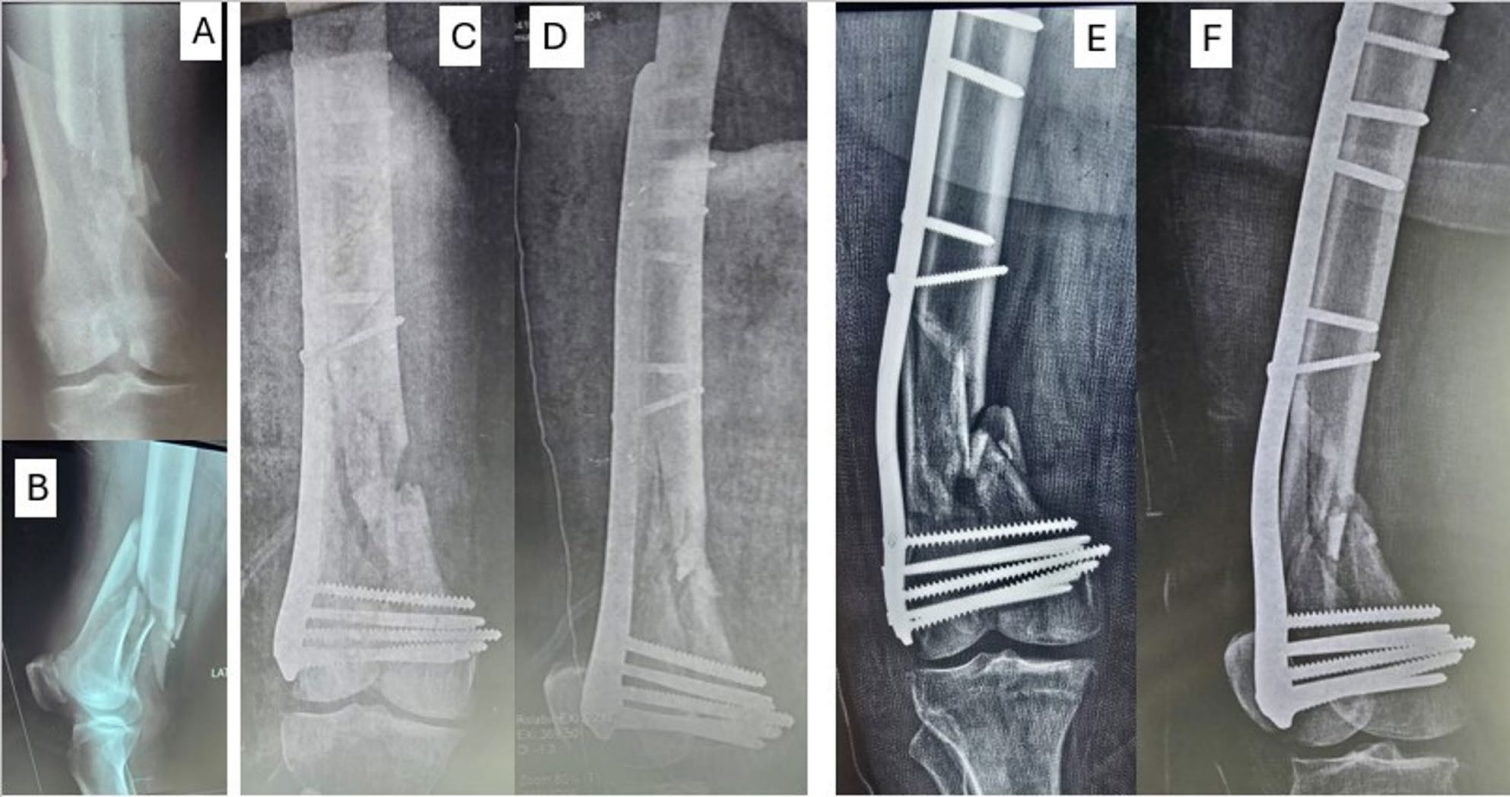
A 75-year-old male patient with a right comminuted distal femur fracture with intraarticular extent **A**, **B** was fixed by a single lateral column plate **C**, **D** and sustained varus nonunion and plastic deformation of the plate 11 weeks after the primary surgery **E**, **F**

The mean follow-up was 19.59 ± 6.38 months (range from 12 to 34 months) with a minimum follow-up of one year; patients were followed through regular visits with functional outcomes recorded at the one-year visit for analysis. Overall, union was successfully achieved in all patients (after dual plate construct application or second-stage surgery), with substantial graft consolidation observed at the fracture site. The clinical union was achieved at an average of 18.4 weeks, while radiological union was slightly delayed, averaging 20.47 weeks. The mean ± SD of the initiation of weight-bearing was 20.5 ± 1.17 weeks. The ROM ranged from 70 to 100° with a mean ± SD of 90.17 ± 10.3**° (**Table 1**)**. (Figures 3, 4 and 5)

**Table 1:** Functional outcomes

Clinical time for union (weeks)	*n* = 30
	18.4 ± 1.83
Radiological Time for union (weeks)	20.47 ± 1.11
Weight-bearing initiation time (weeks)	20.5 ± 1.17
ROM (°)	90.17 ± 10.3

Data are presented as mean ± SD, ROM: range of motion

Most patients (66.67%) achieved an excellent outcome, and 20% had a good result. Only a small proportion had fair (6.67%) or poor (6.67%) outcomes **(**Table 2**)**.

**Table 2:** Neer scoring system

	*n* = 30
Neer scoring system	
Poor	2 (6.67%)
Fair	2 (6.67%)
Good	6 (20%)
Excellent	20 (66.67%)

Data are presented as number (percentage %)

Notably, there were no cases of malunion or nonunion. However, delayed union and infection were each reported in 3 patients (10%), with an overall complication rate of 20%. Patients with delayed union had an autologous bone graft, and union was achieved in the 3 patients. All three patients had open fractures at the primary presentation, and time for union was calculated from the bone grafting surgery. Two superficial wound infections responded to antibiotic therapy, while one patient had a deep-seated infection and refused any further treatment with periods of antibiotic suppression therapy. None of the patients who had complications achieved excellent results, and complications badly affected the outcomes. No patient had revision of fixation during the follow-up period.

A statistically significant relationship is observed regarding both age and BMI with functional outcome (P value < 0.001). Patients with excellent outcomes were significantly younger (mean age 24.9 years) and had a lower BMI (24.31 kg/m²) compared to those with poorer outcomes, who were older (≥ 55 years) and had higher BMIs (≥ 28.7 kg/m²). These findings suggest that younger age and lower BMI are positively associated with better functional recovery. Non-obese patients, especially younger ones, were easy to approach and manipulate the fracture, and shorter surgical times were observed. **(**Table 3**)**

**Table 3:** Relationship between Neer scoring system and other parameters

Age (years)	Poor (*n* = 2)	Fair (*n* = 2)	Good (*n* = 6)	Excellent (*n* = 20)	*P* value
	60 ± 0	55 ± 7.07	42.83 ± 11.84	24.9 ± 8.88	< 0.001*
Sex					
Male	1 (50%)	2 (100%)	3 (50%)	14 (70%)	0.552
Female	1 (50%)	0 (0%)	3 (50%)	6 (30%)	
BMI (kg/m^2^)	28.7 ± 1.56	29.75 ± 0.07	27.72 ± 2.43	24.31 ± 1.95	< 0.001*

Data are presented as mean ± SD or number (percentage %), BMI: body mass index, *: significant as P value ≤ 0.05

Regarding sex, there was no significant association with Neer scores (P value = 0.552), indicating that male and female patients had comparable functional outcomes overall. (Table 3)

## Discussion

DFF, particularly those resulting in nonunion or complex primary fixation, present significant challenges in orthopedic surgery because of their complex biomechanical environment and limited bone stock for fixation [6, 7, 16]. The results of this study, which assessed the effectiveness of distal femoral DP fixation as a salvage method in such instances, showed that most patients had excellent functional recovery and a high union rate with an overall complication rate of 20%.

This study found clinical union in an average of 18.4 weeks and radiological union by 20.47 weeks. These union times are consistent with results reported in a systematic review by Lodde et al. [5], who found that DP of femoral fractures achieved high healing rates, with a fracture healing rate of 88.0% in DFF and 98.5% in non-unions (193 non-unions were included, and fracture healing was achieved in 190 cases). Similarly, Tripathy et al. [1] reported that DP results in adequate union in comminuted metaphyseal and articular fractures of the distal femur, with good to outstanding outcomes observed in 55–75% of patients.

Although 10% of the patients in this study experienced delayed union, none of them suffered from malunion or nonunion. These results are consistent with research using single lateral plating, which has been shown to have nonunion and late union rates as high as 12.5% and 33.3%, respectively [1]. The improved stability given by DP, especially in cases with low bone quality or comminution, is hypothesized to minimize micromotion and improve the mechanical environment for healing. Biomechanical studies support this, demonstrating increased stiffness and load-sharing capacity in DP constructs compared to single plating, especially under axial and torsional forces [4, 7, 17].

The excellent Neer outcomes observed in 66.67% of our cases further support the effectiveness of this fixation technique. The positive correlation between younger age and lower BMI with better functional outcomes aligns with findings from a multicenter study by Kuwahara et al. [18], who discovered that a low body mass index (BMI) was an independent risk factor for impaired postoperative functional recovery and increased mortality in DFF among elderly cases.

Similarly, obesity has been associated with delayed bone healing and higher complication rates. A study by Bryant et al. [19] reported that elevated BMI increases the risk of longer hospitalization and systemic complications, with obese patients experiencing more comorbidities and complications compared to normal-weight patients.

The average operating time (161.17 ± 29.23 min) and intraoperative blood loss (526.67 ± 164.05 ml) were not consistent with Nam et al. [20], who discovered that the average operation time and intraoperative bleeding were 81 min (66–92 min) and 467 ml (338–581 ml) in the single-plate group and 110 min (95–120 min) and 573 ml (381–657 ml) in the dual-plate group, respectively.

This increase is expected given the technical demands of DP, including soft tissue handling, exposure for medial plating, and implant positioning. Despite the extended surgical duration, the mean hospital stay of 2.83 days suggests that postoperative recovery was not significantly prolonged, likely because of proper perioperative care and early mobilization protocols.

At 10% for both infection and delayed union, the complication incidence was comparatively modest. Compared to rates reported in some publications, this is noticeably better. For example, DP has been associated with infection rates ranging from 0% to 16.7%, compared with 3.6% to 8.5% for lateral plating alone [3]. The reduced complication rate in this study could be attributed to strict aseptic technique, selection of fit patients (exclusion of immunocompromised or infected cases), and the use of modern locking compression plates (LCPs), which reduce stress shielding and promote biological healing.

Not all studies agree with the superiority of DP. Some studies have raised concerns regarding the use of DP, particularly in terms of soft tissue compromise and potential for devascularization [8]. However, in this study, careful surgical dissection and limited soft tissue stripping were prioritized to minimize such risks. There was just one documented instance of a profound infection or no wound breakdown.

On comparing the dual plating cases with that of single plating, Shah et al. [21] found the dual plating group superior to the single plating group both in terms of functional outcome and the extent of complications. Also, the dual plating group has better knee ROM. The medial and lateral locked plating technique demonstrates a higher union rate, with possibly lower rates of revision surgery, compared to a single lateral plate in highly comminuted distal femur fractures. Wright et al. found DP constructs provided stiffer fixation than plate-nail constructs in this biomechanical study of extra-articular distal femur fractures [7, 11, 12].

Furthermore, a study by Bologna et al. [22] found that DP resulted in higher union rates in comminuted distal femur fractures than single plate fixation, with no non-union or delayed unions noted in the DP group. This gives support to the use of dual plating in complex fracture situations.

This study has several important limitations. First, the relatively small sample size and the absence of a comparative cohort treated with alternative fixation strategies—such as isolated lateral plating—limit the strength of the conclusions and reduce the ability to perform robust comparative analyses. Additionally, the heterogeneity of the included cases, encompassing both complex fractures and established nonunion, introduces variability that may influence outcomes and should be considered when interpreting the findings. This lack of long-term follow-up further limits the evaluation of late sequelae, which are clinically significant considerations in distal femoral reconstruction and include implant fatigue failure, increasing deformity, osteoporosis, stress shielding, and delayed postoperative knee stiffness.

Despite these limitations, the results highlight important trends that support the potential advantages of dual plating in select patient populations. However, bigger, more uniform cohorts with standardized injury patterns and well-defined fixation techniques should be part of future studies. In particular, prospective controlled or randomized comparative studies are required to determine the relative effectiveness of dual plating in comparison to alternative constructs and to produce more conclusive, broadly applicable clinical practice recommendations.

## Conclusion

Distal femur dual plating is a viable salvage option for complex primary fixation of complex distal femur fractures. Good to excellent outcomes can be achieved with a low complication rate, especially in patients with normal BMI and younger than 55 years of age.

## Data Availability

Available on request, the corresponding author is responsible for data availability (ITB).

## Declarations

The study adhered to the Declaration of Helsinki.

## References

[1] Tripathy SK, Mishra NP, Varghese P, Panigrahi S, Purudappa PP, Goel A, Mishra NP, Varghese P, Panigrahi S, Purudappa PP, Goel A, et al. RK SenIndian J Orthop. 2022;56(2):183–207. 10.1007/s43465-021-00489-0. 10.1007/s43465-021-00489-0 PMC8789962 35140850

[2] DeKeyser GJ, Hakim AJ, O’Neill DC, Schlickewei CW, Marchand LS, Haller JM. Biomechanical and anatomical considerations for dual plating of distal femur fractures: a systematic literature review. Arch Orthop Trauma Surg. 2022;142(10):2597–609. 10.1007/s00402-021-03988-9 34097123 10.1007/s00402-021-03988-9

[3] Thorne TJ, Dong W, Higgins TF, Rothberg DL, Haller JM, Marchand LS. Primary repair of complete quadriceps tendon rupture with extensor mechanism deficit. JBJS Essent Surg Techniques. 2024;14(3):e23. https://journals.lww.com/jbjsest/fulltext/2024/06000/dual_plating_of_distal_femoral_fractures.7.aspx 10.2106/JBJS.ST.23.00045 PMC11415094 39314211

[4] Oun A, Sabra HK, Abdelaziz O, Elhois IS, Saleh AO, Hemdan K, et al. Dual vs. single plating in distal femoral fractures: a systematic review and meta-analysis. J Orthop Surg Res. 2025;20(1):923. 10.1186/s13018-025-06309-7. :1. 24 ottobre 2025. 41137092 10.1186/s13018-025-06309-7 PMC12553283

[5] Lodde MF, Raschke MJ, Stolberg-Stolberg J, Everding J, Rosslenbroich S, Katthagen JC. Union rates and functional outcome of double plating of the femur: systematic review of the literature. Arch Orthop Trauma Surg. 2022;142(6):1009–30. 10.1007/s00402-021-03767-6 33484313 10.1007/s00402-021-03767-6 PMC9110521

[6] Barwar N, Gargi G, Rai A, Elhence A, Banerjee S, Gahlot N. Dual plating in the management of nonunion complex distal femur fractures following lateral locked plate fixation: radiological and functional outcomes of a prospective study. J Trauma Injury 1 giugno. 2025;38(2):125. 10.20408/jti.2024.0054 10.20408/jti.2024.0054 PMC12229817 40571955

[7] Xu W, Lin W, Liu H, Xiong Y, Zhang J, Wu J. Comparison of combined femoral nail and plate fixation versus dual plate fixation in the treatment of AO/OTA 33C distal femoral fractures. Sci Rep. 2025;15(1):28911. . 10.1038/s41598-025-14999-6 40775521 10.1038/s41598-025-14999-6 PMC12331908

[8] Rollick NC, Gadinsky NE, Klinger CE, Kubik JF, Dyke JP, Helfet DL, Wellman DS. The effects of dual plating on the vascularity of the distal femur. Bone Joint J. 2020;102(4):530–8. 10.1302/0301-620X.102B4 32228080 10.1302/0301-620X.102B4.BJJ-2019-1776

[9] El Beaino M, Morris RP, Lindsey RW, Gugala Z. Biomechanical evaluation of dual plate configurations for femoral shaft fracture fixation. Biomed Res Int 1 gennaio. 2019;2019(1):5958631. 10.1155/2019/5958631 10.1155/2019/5958631 PMC6512036 31183369

[10] DeKeyser GJ, Hakim AJ, O’Neill DC, Schlickewei CW, Marchand LS, Haller JM. Biomechanical and anatomical considerations for dual plating of distal femur fractures: a systematic literature review. Arch Orthop Trauma Surg. 2022;142(10):2597–609. 10.1007/s00402-021-03988-9 34097123 10.1007/s00402-021-03988-9

[11] Steimle J, Lohmeyer JL, Taylor B, DeGenova DT. The Role of Adjunctive Medial Plating in Distal Femur Fractures and Distal Femur Fractures Nonunions. Cureus. 2025;17(2):e78538–78538. 40062069 10.7759/cureus.78538 PMC11888361

[12] Wright DJ, Desanto DJ, McGarry MH, Lee TQ, Scolaro JA. Supplemental Fixation of Supracondylar Distal Femur Fractures: A Biomechanical Comparison of Dual-Plate and Plate-Nail Constructs. J Orthop Trauma 1 agosto. 2020;34(8):434–40. 10. 1097/BOT.0000000000001749 PubMed PMID: 32032183. 10.1097/BOT.0000000000001749 32032183

[13] Schütz M, Müller M, Regazzoni P, Höntzsch D, Krettek C, Van der Werken C, Haas N. Use of the less invasive stabilization system (LISS) in patients with distal femoral (AO33) fractures: a prospective multicenter study. Arch Orthop Trauma Surg. 2005;125(2):102–8. 10.1007/s00402-004-0779-x 15688230 10.1007/s00402-004-0779-x

[14] Henderson CE, Kuhl LL, Fitzpatrick DC, Marsh JL. Locking plates for distal femur fractures: is there a problem with fracture healing? J Orthop Trauma. 2011;25:S8–14. https://journals.lww.com/jorthotrauma/fulltext/2011/02001/locking_plates_for_distal_femur_fractures__is.3.aspx 21248560 10.1097/BOT.0b013e3182070127

[15] CHARLES S NEER II, Grantham SA, Shelton ML. Supracondylar fracture of the adult femur: a study of one hundred and ten cases. JBJS. 1967;49(4):591–613. 6025996

[16] Siddiqui YS, Mohd J, Abbas M, Gupta K, Khan MJ, Istiyak M. Technical difficulties and mechanical failure of distal femoral locking compression plate (DFLCP) in management of unstable distal femoral fractures. Int J Burns Trauma. 2021;11(1):9. . https://pmc.ncbi.nlm.nih.gov/articles/PMC8012867/33824780 PMC8012867

[17] Cheung ZB, Nasser P, Iatridis JC, Forsh DA. Orthogonal plating of distal femur fractures: A biomechanical comparison with plate-nail and parallel plating constructs. J Orthop. 2023;37:34–40. . https://www.sciencedirect.com/science/article/pii/S0972978X23000351 36974099 10.1016/j.jor.2023.02.003 PMC10039308

[18] Kuwahara Y, Takegami Y, Tokutake K, Yamada Y, Komaki K, Imagama S. Low body mass index is a risk factor for increased post-operative mortality and poor functional improvement in distal femur fractures among patients aged over 65: A multicentre (TRON) study. J Orthop Sci. 2023;28(3):631–6. . https://www.sciencedirect.com/science/article/pii/S0949265822000276 35190219 10.1016/j.jos.2022.01.014

[19] Bryant MK, Parrish M, Roy S, Udekwu P, Farrell M, Schinco M, Ganga S. Inferior clinical outcomes after femur fracture in the obese are potentially preventable. Injury. 2019;50(11):2049–54. https://www.sciencedirect.com/science/article/pii/S0020138319304991 31447210 10.1016/j.injury.2019.08.026

[20] Nam DJ, Kim MS, Kim TH, Kim MW, Kweon SH, Kim MS, Kim TH, Kim MW. 2022•Springer 1 dicembre. 2022;17(1):55. 10.1186/s13018-022-02944-6. SH KweonJournal of Orthopaedic Surgery and ResearchPubMed PMID: 35093125.

[21] Shah A, Agarwal S, Nagaich A. Comparative study of single vs. dual plating in distal femur fracture. J Bone Joint Dis gennaio. 2024;39(1):1–8. 10.4103/jbjd.jbjd_27_23.

[22] Bologna MG, Claudio MG, Shields KJ, Katz C, Salopek T, Westrick ER. Dual plate fixation results in improved union rates in comminuted distal femur fractures compared to single plate fixation. J Orthop. 2020;18:76–9. https://www.sciencedirect.com/science/article/pii/S0972978X19304179 32189888 10.1016/j.jor.2019.09.022 PMC7068022

